# Is FOP Nutrition Label Nutri-Score Well Understood by Consumers When Comparing the Nutritional Quality of Added Fats, and Does It Negatively Impact the Image of Olive Oil?

**DOI:** 10.3390/foods10092209

**Published:** 2021-09-17

**Authors:** Morgane Fialon, Jordi Salas-Salvadó, Nancy Babio, Mathilde Touvier, Serge Hercberg, Pilar Galan

**Affiliations:** 1 Sorbonne Paris Nord University, Inserm U1153, Inrae U1125, Cnam, Nutritional Epidemiology Research Team (EREN), Epidemiology and Statistics Research Center—University of Paris (CRESS), 93017 Bobigny, France; m.touvier@eren.smbh.univ-paris13.fr (M.T.); s.hercberg@eren.smbh.univ-paris13.fr (S.H.); p.galan@uren.smbh.univ-paris13.fr (P.G.); 2 Consorcio CIBER, M.P. Fisiopatología de la Obesidad y Nutrición (CIBEROBN), Instituto de Salud Carlos III, 28029 Madrid, Spain; jordi.salas@urv.cat (J.S.-S.); nancy.babio@urv.cat (N.B.); 3 Universitat Rovira i Virgili, Departament de Bioquímica i Biotecnologia, Unitat de Nutrició Humana, 43201 Reus, Spain; 4 Institut d’Investigació Sanitària Pere Virgili (IISPV), 43204 Reus, Spain; 5 Public Health Department, Avicenne Hospital, Assistance Publique des Hôpitaux de Paris (AP-HP), 93017 Bobigny, France

**Keywords:** olive oil, Nutri-Score, added fats, nutritional quality, lobbying

## Abstract

It has been suggested that the current ranking of olive oil by Nutri-Score (C) is not in line with its nutritional quality and could have a negative impact on the sales and consumption of olive oil, especially compared to other added fats with lower nutritional qualities One of the objectives of this study is to investigate consumers’ understanding of Nutri-Score when comparing the nutritional quality of added fats, and to test whether or not Nutri-Score has a negative impact on the image and the choice of olive oil in a sample of Spanish consumers. A cross-sectional study of 486 Spanish adults (mean age ± SD: 45.8 ± 14.0 years; 48.6% women) was conducted. Subjects were recruited through a web panel provider to participate in an online questionnaire. Almost 80% of participants declared that Nutri-Score was useful for recognizing the differences in nutritional quality between the eight added fats presented in the study; 89.1% rightly identified that olive oil was among the added fats with the best nutritional quality (vs. 4.1% for rapeseed oil (Nutri-Score C), and less than 3% for other added fats (Nutri-Score D or E)). When asked about which added fat they would buy more frequently, 86.2% of participants selected olive oil. Then, after being reminded that among added fats, the Nutri-Score C given to olive oil was the best grade, a majority of participants declared that they would keep consuming olive oil as much as before (71.4%). Finally, almost 78% of participants thought that Nutri-Score should be displayed on olive oil. In conclusion, the results of our study suggest that displaying Nutri-Score on olive oil was well accepted and understood by a large majority of participants who appeared to accept the current ranking of the Nutri-Score for olive oil (Nutri-Score C).

## 1. Introduction

The Nutri-Score is a summary, graded, color-coded front-of-pack nutrition label (with five categories from dark green/A to dark orange/E) intended to inform consumers, in a simple and understandable way, of the overall nutritional quality of foods in order to help them compare products and make healthier choices at the point of purchase [1]. Since it was proposed in 2014 in France by independent academic researchers, the Nutri-Score has been the subject of numerous attacks by agro-food industry lobbies, which have been reluctant to provide transparency on the nutritional quality of their food products and, therefore, have acted to prevent its deployment in France [2] and now in Europe [3]. For four years, major food lobby groups developed various strategies to try to block its application [4,5]. They denied or questioned the results of the abundant scientific studies that demonstrated the effectiveness of Nutri-Score compared to other existing graphical formats [6]. In 2020, the large multi-country FOP-ICE study in 12 European countries including Spain [7,8] showed that the Nutri-Score appeared as the best scheme to help participants identify healthier food products compared to other front-of-pack nutrition labels (FoPLs). Several studies also highlighted Nutri-Score’s ability to discriminate products across the same food category as well as its consistency in regard to European national dietary guidelines [9,10].

Thanks to the mobilization of several societal actors (scientists, health professionals, consumer and patient associations, etc.) who emphasized the relevance of Nutri-Score as a public health tool and recommended its implementation, Nutri-Score was finally adopted in France in October 2017, on a voluntary basis due to European regulations. Although it was also officially adopted in Belgium, Germany, Luxembourg, Spain, the Netherlands and Switzerland, opponents are still very active at the European level to prevent its adoption as a single and mandatory logo in 2022 within the framework of the Farm to Fork strategy programmed by the European Commission [11].

Moreover, some agricultural production sectors, especially the processed meat, cheese and olive oil industry expressed their opposition to Nutri-Score, arguing it does not classify their products sufficiently well [12,13]. With respect to processed meat and cheese, many products are classified as D and E due to their high content in saturated fatty acids and salt, as well as their high caloric density. Nutri-Score’s grades for these products are aligned with nutritional recommendations, since official food-based dietary guidelines in most occidental countries consistently recommend limiting their consumption. A large majority of scientists, even in Mediterranean countries where cold cuts and cheese are a part of the culinary patrimony, consider the Nutri-Score classification of these products to be relevant, due to their poor nutritional composition. However, some concerns have been raised, especially in Spain, regarding the classification of added fats and especially olive oil, whose consumption is recommended by public health authorities as one of the preferred added fats for cooking or seasoning within the framework of the Mediterranean diet. Indeed, some scientists felt that assigning it a Nutri-Score C—in the middle range of the classification—even if it is the best score among added fats, could have a negative impact on its perception as an important component of a healthy diet.

Besides the scientific discussion, producers and industrialists in the olive oil sector in Spain, Italy and Greece also considered that ranking it Nutri-Score C could have a negative impact on exports and internal sales, potentially lowering its consumption in favor of other added fats with lower nutritional quality [14,15]. Even if olive oil is classified in the best Nutri-Score category for added fats—as no added fats are ranked Nutri-Score A or B—lobbies have stoked the flames and have suggested that Nutri-Score would actually oppose nutritional recommendations.

The objective of this study is to investigate consumers’ understanding of Nutri-Score to compare the nutritional quality of added fats and to test if Nutri-Score could negatively impact the image and the choice of olive oil among a sample of Spanish consumers.

## 2. Materials and Methods

### 2.1. Design, Population Study and Procedures

A cross-sectional study was conducted of a total of 486 Spanish adults recruited through a web panel provider (Pureprofile), applying quotas for sex (50% of women), age and educational level to ensure representativeness of the Spanish general adult population.

**Figure 1:**
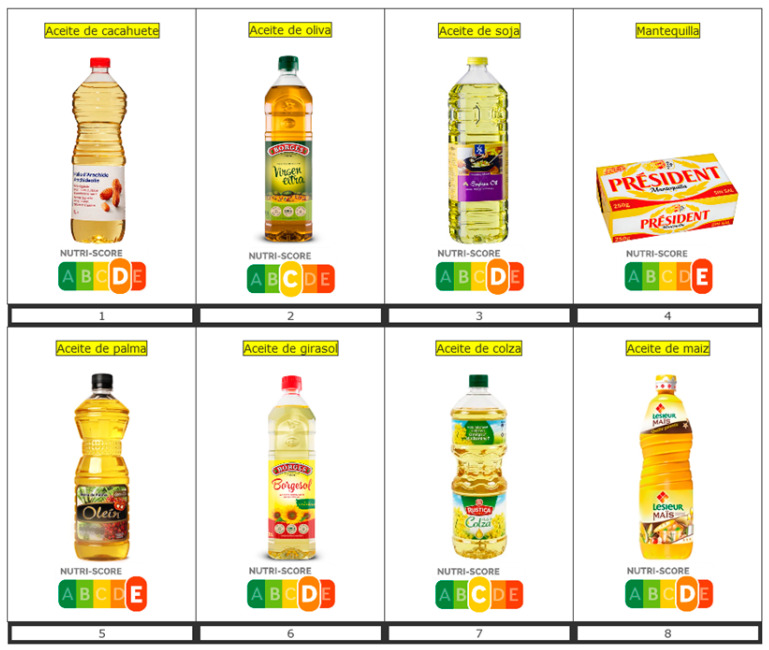
Pictures of the different vegetable oils and butter tested with their corresponding Nutri-Score from C to E.

Participants were invited to respond to an online questionnaire that was presented in Spanish. At the beginning of the survey, participants were asked to provide information on sex, age, educational level, size of household, self-estimated diet quality and self-estimated level of nutrition knowledge. Then, participants had to respond to questions pertaining to perception and understanding of Nutri-Score. The present study focuses specifically on the questions related to perceptions of added fat. Pictures of seven vegetable oils and butter, with their corresponding Nutri-Score, were presented to participants (Figure 1).

Participants were asked to answer specific questions about the usefulness of Nutri-Score to differentiate the nutritional quality of the presented added fats using a 4-point Likert scale (ranging from “Strongly disagree” to “Strongly agree” with an “I don’t know” option). Additionally, they had to specify the added fat they considered to have the best nutritional quality, and which one they would buy more frequently. Then, questions were asked about how Nutri-Score would impact their future purchases of olive oil compared to their current ones, and if they were in favor of the Nutri-Score being displayed on olive oil packaging.

### 2.2. Statistical Analysis

Socio-demographic characteristics were controlled statistically for the items “Nutri-Score helps me differentiate these added fats in terms of nutritional quality” (answers converted in a binary outcome (yes/no) and participants who responded “I don’t know” were excluded for this analysis) and “Nutri-Score should be displayed on olive oil”. The variables included as covariates were: age of the participant (continuous), sex, educational level, presence of children aged 13 years old or lower in the household, self-perceived diet quality and self-rated nutritional knowledge. Multi-adjusted logistic regression analyses were conducted to compare participants who agreed that Nutri-Score helped them differentiate the proposed added fats in terms of nutritional quality and those who disagree, as well as participants who thought Nutri-Score should be displayed on olive oil packaging and those who did not. Statistical tests were two-sided. A *p*-value below 0.05 was considered statistically significant. All analyses were completed using R software.

## 3. Results

Individual characteristics of the study sample are described in Table 1. The present study included 486 participants, of whom 51.4% were men, 32.5% were individuals over 54 years old, and 38.1% had a university degree. The mean age ± SD was: 45.8 ± 14.0 years. Among all participants, 13.6% declared having a very or mostly unhealthy diet quality, and 58.5% declared having no or little knowledge about nutrition.

**Figure 2:**
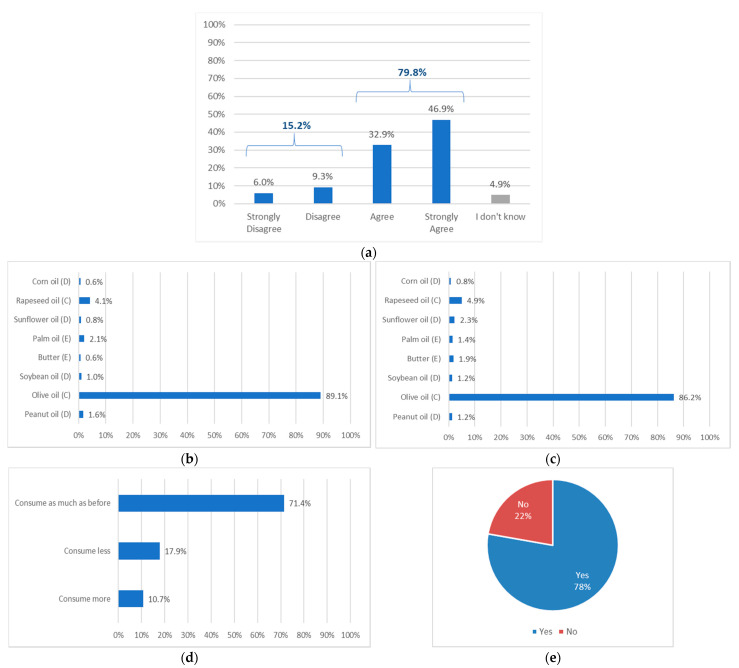
(**a**) Participants’ opinions on the statement “Nutri-Score helps me differentiate the nutritional qualities of the added fats presented”; (**b**) Participants’ answers when selecting the added fat considered to have the best nutritional qualities; (**c**) Participants’ answers when selecting the added fat that they would buy the most frequently; (**d**) Participants’ answers regarding the effect of Nutri-Score C-rated-olive oil on their olive oil consumption; (**e**) Participants’ answers to the question “Do you think olive oil should be labelled with Nutri-Score?”.

Overall, 79.8% of the participants declared that Nutri-Score was useful for recognizing differences in nutritional quality between the eight presented added fats (Figure 2a). Using Nutri-Score, 89.1% of the study population considered olive oil to be the healthiest among the proposed added fats (vs. 4.1% for rapeseed oil, 1.6% for peanut oil, 2.1% for palm oil and around 1% for the other added fats, Figure 2b). A large majority (86.2%) declared that they would buy olive oil more frequently (vs. 4.9% for rapeseed oil and less than 3% for the others added fats, Figure 2c).

After providing the participants with the information of Nutri-Score C being the best grade among added fats, 10.7% of participants declared that they would consume more olive oil than they currently do and 71.4% replied that they would consume it as much as before (Figure 2d). Finally, 77.8% considered that Nutri-Score should be displayed on olive oil packaging (Figure 2e).

The results of multiple logistic regression analysis (Table 2 and Table 3) showed that the tendency to agree with the item “Nutri-Score helps me differentiate these added fats in terms of nutritional quality” increased proportionally with age (OR = 1.02, CI = 1.00–1.04, *p* = 0.03) and the fact of having children (OR = 2.15, CI = 1.19–4.03, *p* = 0.01) compared to the tendency to disagree. Agreeing that Nutri-Score should be displayed on olive oil was significantly associated with having children (OR = 1.82, CI = 1.10–3.10, *p* = 0.02). While no significant association was found with self-estimated nutrition knowledge in this study, it can be noted that the interest in front-of-pack labels among Polish consumers was shown to be significantly predicted by self-rated nutrition knowledge in 2020 [16].

## 4. Discussion

This study provides new insights concerning the perception and understanding of Nutri-Score in the case of added fats, specifically olive oil, in light of the controversy surrounding Nutri-Score among some scientists and powerful lobbies, particularly in Mediterranean countries such as Spain. They consider that the C classification of olive oil has a negative impact on the image of olive oil and, consequently, on its sales (in a context where olive oil is one of the main exports of Mediterranean countries). In fact, Nutri-Score is in line with the model of the Mediterranean diet that invites consumers to favor olive oil in their consumption of added fats, especially in countries where it is part of the culinary culture, while insisting on the importance of avoiding excess fat in general, whatever they are. Nutri-Score rates added fats consistently with nutritional and public health recommendations that encourage consumers to prefer vegetable oils over animal fats (such as butter) and to strongly favor olive oil among vegetable oils. Ranking olive oil Nutri-Score C is, then, fully consistent with nutritional recommendations, as it is the best possible grade for vegetable oils (together with rapeseed oil and walnut oil), and is better than soybean, sunflower or corn oil (classified D), coconut or palm oil (classified E) and butter (classified E). No oil is classified A or B. It is, therefore, clear that this classification is, in fact, favorable to olive oil, highlighting its nutritional quality. As a result, if consumers want to choose a vegetable oil, they will easily see on supermarket shelves that olive oil is the best ranked product compared to other oils (along with rapeseed and walnut oil), thanks to Nutri-Score. Our results show that a large majority of Spanish consumers participating in this study considered the Nutri-Score to be useful in helping them recognize the differences in nutritional quality between added fats; and that consumers would most often purchase olive oil. Even if rapeseed oil is also classified Nutri-Score C, olive oil was preferred by Spanish consumers (only 4.1% responded that rapeseed oil was the added fat with the best nutritional quality, in line with its Nutri-Score C ranking). No major negative impact on the intention of participants to buy olive oil in the future was observed; if 17.9% considered they would consume less olive oil than before, 10.7% declared they would increase their consumption, and the large majority (71.4%) declared they were planning to consume olive oil as much as they currently do. It is interesting to note that these favorable results for olive oil were observed with a simple communication about Nutri-Score during the survey (one screen giving some background on the scheme at the beginning of the questionnaire) but without any emphasis on the health benefits of olive oil.

Therefore, our results show that Nutri-Score does not appear to negatively impact the perception of olive oil by Spanish consumers and does not affect their intended behavior in relation to the recommendations to favor olive oil among added fats. This recommendation is linked to the epidemiological studies, especially meta-analysis and interventional trials, which consistently show a beneficial association between olive oil consumption as part of a Mediterranean diet and health, particularly in relation to the prevention of cardiovascular diseases [17,18,19]. Some other meta-analyses published in the scientific literature [20,21,22] also support the health benefits of rapeseed and walnut oils in the prevention of cardiovascular diseases (as their consumption is associated with healthier blood lipid profiles), so many countries recommend favoring olive oil, rapeseed oil and walnut oil among vegetable oils.

Our results are consistent with the data published by a Spanish retailer that adopted Nutri-Score two years ago and analyzed the sales evolution during this period [23]. The data shows that with the arrival of Nutri-Score, consumers have not stopped choosing olive oil as a usual product in their shopping basket and market shares for olive oils have not been negatively affected.

In the heated debate in Spain over Nutri-Score, pressure from the olive oil lobbies raised the issue of the exclusion of olive oil from the Nutri-Score application. The same strategy has also been requested by other agricultural sectors such as ham, cheese and other traditional food manufacturers, who consider that Nutri-Score penalizes traditional foods. The arguments used by these stakeholders stress the fact that olive oil, cold cuts and cheese are part of the culinary and gastronomic culture of the region or country, thus confusing two different dimensions of a food product—namely its traditional manufacturing process and its nutritional composition—which can be misleading for consumers. It is interesting to note that our results do not support such a strategy for olive oil, as more than 3/4 of our study participants supported the use of Nutri-Score on olive oil packaging.

Finally, results of our study suggest that displaying Nutri-Score on olive oil is well accepted and understood by a large majority of participants who seemed to understand that the letter C was the best rank an added fat could get. An adapted communication highlighting the health benefits of olive oil consumption, especially the virgin olive oil varieties [24,25], may be necessary to reinforce this information and avoid misunderstanding among the small percentage of consumers who could have some difficulties in understanding how to use Nutri-Score.

In addition, a Scientific Committee set up at European level, composed of independent experts from the seven countries that adopted Nutri-Score, is in charge of updating the Nutri-Score on scientific bases. In the framework of this process, the classification of added fats and olive oil will necessarily be investigated on the basis of their nutritional composition and health effects.

## Figures and Tables

**Table 1:** Individual characteristics of the study sample (N = 486).

	N (%)
**Sex**	
Men	250 (51.4)
Women	236 (48.6)
**Age, years**	
18–34	115 (23.7)
35–54	213 (43.8)
55–80	158 (32.5)
**Educational level**	
Primary education	13 (2.7)
Secondary education	42 (8.6)
Trade certificate ^1^	246 (50.6)
University undergraduate degree	139 (28.6)
University postgraduate degree	46 (9.5)
**Number of persons in household**	
Total	mean = 2.91 (SD = 1.19) Range: 1 to 9
Number of persons ≤ 13 years	mean = 0.44 (SD = 0.70) Range: 0 to 4
**Self-estimated diet quality**	
I eat a very unhealthy diet	1 (0.2)
I eat a mostly unhealthy diet	65 (13.4)
I eat a mostly healthy diet	371 (76.3)
I eat a very healthy diet	49 (10.1)
**Self-estimated nutrition knowledge**	
I do not know anything about nutrition	27 (5.6)
I am not very knowledgeable about nutrition	257 (52.9)
I am somewhat knowledgeable about nutrition	187 (38.5)
I am very knowledgeable about nutrition	15 (3.1)

^1^ Trade certificate corresponds to a career-specific certificate earned in a maximum of two years.

**Table 2:** Association between the variable “Nutri-Score helps me differentiate these added fats in terms of nutritional quality” (participants who did not agree as reference, N = 74) and socio-demographic characteristics using multi-adjusted logistic regression analyses (N = 462).

N = 462 *(Participants Who Responded « I Don’t Know » (N = 24) Were Excluded from the Analysis)*		
**Variables**	OR (95% CI)	*p*-Value
**Age (continuous)**	1.02 (1.00–1.04)	0.03
**Sex**		
**Male**	Ref	
**Female**	1.31 (0.79–2.20)	0.30
**Education**		
**Trade certificate or lower** **^1^**	Ref	
**University degree**	0.62 (0.37–1.05)	0.08
**Has Children**		
**No**	Ref	
**Yes**	2.15 (1.19–4.03)	0.01
**Self-perceived diet quality**		
**Poor diet quality**	Ref	
**Good diet quality**	0.74 (0.30–1.60)	0.47
**Self-rated nutritional knowledge**		
**Little knowledge of nutrition**	Ref	
**Good knowledge of nutrition**	0.73 (0.43–1.24)	0.24

^1^ Trade certificate corresponds to a career-specific certificate earned in a maximum of two years.

**Table 3:** Association between the variable “Do you think olive oil should be labelled with Nutri-Score?” (Participants who answered “no” as reference, N = 108) and socio-demographic characteristics using multi-adjusted logistic regression analyses (N = 486).

N = 486		
**Variables**	OR (95% CI)	*p*-Value
**Age (continuous)**	1.01 (0.99–1.02)	0.54
**Sex**		
**Male**	Ref	
**Female**	0.71 (0.46–1.11)	0.13
**Education**		
**Trade certificate or lower** **^1^**	Ref	
**University degree**	0.69 (0.44–1.09)	0.11
**Has Children**		
**No**	Ref	
**Yes**	1.82 (1.10–3.10)	0.02
**Self-perceived diet quality**		
**Poor diet quality**	Ref	
**Good diet quality**	0.66 (0.30–1.34)	0.27
**Self-rated nutritional knowledge**		
**Little knowledge of nutrition**	Ref	
**Good knowledge of nutrition**	0.65 (0.41–1.03)	0.07

^1^ Trade certificate corresponds to a career-specific certificate earned in a maximum of two years.

## Data Availability

The data used in this study are available on request to the corresponding author.
